# Food Packaging and Bisphenol A and Bis(2-Ethyhexyl) Phthalate Exposure: Findings from a Dietary Intervention

**DOI:** 10.1289/ehp.1003170

**Published:** 2011-03-30

**Authors:** Ruthann A. Rudel, Janet M. Gray, Connie L. Engel, Teresa W. Rawsthorne, Robin E. Dodson, Janet M. Ackerman, Jeanne Rizzo, Janet L. Nudelman, Julia Green Brody

**Affiliations:** 1 Silent Spring Institute, Newton, Massachusetts, USA; 2 Breast Cancer Fund, San Francisco, California, USA; 3 Vassar College, Poughkeepsie, New York, USA; 4 AXYS Analytical Services, Sidney, British Columbia, Canada

**Keywords:** canned foods, diet, endocrine disruptor, exposure, food packaging, intervention design, pharmacokinetics, phthalates, plastics

## Abstract

Background: Bisphenol A (BPA) and bis(2-ethylhexyl) phthalate (DEHP) are high-production-volume chemicals used in plastics and resins for food packaging. They have been associated with endocrine disruption in animals and in some human studies. Human exposure sources have been estimated, but the relative contribution of dietary exposure to total intake has not been studied empirically.

Objectives: To evaluate the contribution of food packaging to exposure, we measured urinary BPA and phthalate metabolites before, during, and after a “fresh foods” dietary intervention.

Methods: We selected 20 participants in five families based on self-reported use of canned and packaged foods. Participants ate their usual diet, followed by 3 days of “fresh foods” that were not canned or packaged in plastic, and then returned to their usual diet. We collected evening urine samples over 8 days in January 2010 and composited them into preintervention, during intervention, and postintervention samples. We used mixed-effects models for repeated measures and Wilcoxon signed-rank tests to assess change in urinary levels across time.

Results: Urine levels of BPA and DEHP metabolites decreased significantly during the fresh foods intervention [e.g., BPA geometric mean (GM), 3.7 ng/mL preintervention vs. 1.2 ng/mL during intervention; mono-(2-ethyl-5-hydroxy hexyl) phthalate GM, 57 ng/mL vs. 25 ng/mL]. The intervention reduced GM concentrations of BPA by 66% and DEHP metabolites by 53–56%. Maxima were reduced by 76% for BPA and 93–96% for DEHP metabolites.

Conclusions: BPA and DEHP exposures were substantially reduced when participants’ diets were restricted to food with limited packaging.

Bisphenol A (BPA) is a high-production- volume industrial chemical used in the manufacture of polycarbonate and other plastic products and epoxy resin–based food can liners. It is present in both canned and plastic-packaged foods sold in the United States (Schecter et al. 2010). Exposure is widespread, with detectable levels in urine samples from > 90% of the U.S. population (Calafat et al. 2008). A wide body of evidence from *in vitro*, animal, and epidemiological studies indicates the potential for BPA-induced endocrine disruption in a number of organ systems. The uses, exposure, and health effects of BPA have been reviewed elsewhere [National Toxicology Program Center for the Evaluation of Risks to Human Reproduction (NTP-CERHR) 2008; Food and Agriculture Organization of the United Nations/World Health Organization 2010].

Phthalates are another common class of endocrine-disrupting chemicals (EDCs) produced in high volumes and widely used in consumer goods, including food packaging (European Food Safety Authority 2005; Fromme et al. 2007; NTP-CERHR 2006; Wormuth et al. 2006). This family of EDCs includes higher-molecular-weight phthalates such as bis(2-ethylhexyl) phthalate [DEHP; a common polyvinyl chloride (PVC) additive], dibutyl phthalate (DBP), and butyl benzyl phthalate (BBP), and also lower-molecular-weight phthalates such as dimethyl phthalate (DMP) and diethyl phthalate (DEP), which is commonly used as a solvent for fragrance. All of these are used in food packaging. The higher-molecular-weight phthalates DEHP, DBP, and BBP are identified as EDCs based on inhibition of testosterone synthesis and effects on the developing male reproductive system in rodents, whereas the lower-molecular- weight phthalates DEP and DMP did not induce these effects (Gray et al. 2000). Some epidemiological evidence shows associations between urinary excretion of phthalate metabolites and effects on the developing male reproductive system (Swan 2008), male hormone levels and semen quality (Hauser 2008; Meeker et al. 2007, 2009), and neurobehavioral end points (Engel et al. 2009).

Exposure estimates based on food, air, dust, and consumer product concentrations and intake rates indicate that diet is likely to be a major source of exposure for BPA and DEHP (Fromme et al. 2007; Lakind and Naiman 2010; NTP-CERHR 2006, 2008) and an important source of exposure to BBP and DBP (NTP-CERHR 2003a, 2003b; Wormuth et al. 2006). Diet is expected to account for only a small fraction of exposure to DMP and DEP, which are predominantly from consumer product sources (Itoh et al. 2007; Wormuth et al. 2006). However, empirical data to verify these estimates are limited.

Better information about exposure sources, such as the role of diet, is needed to provide reliable information about opportunities to reduce exposure. Many individuals seek guidance to avoid exposures as a precaution while health effects remain under study. In addition, the U.S. Food and Drug Administration (FDA) recently announced its support for “reasonable steps” by the agency to reduce BPA exposure (FDA 2010).

The contribution of different sources and the effectiveness of exposure reduction strategies can be efficiently evaluated through longitudinal studies of small numbers of participants in interventions designed to alter exposure. BPA and phthalates are suited to this design because they have short biological half-lives, noninvasive exposure biomarkers, and sources that can be modified by individual behaviors. The value of this design has been demonstrated in studies that showed an increase in urinary BPA in students using polycarbonate drinking water bottles (Carwile et al. 2009); reductions in urinary pesticide metabolites in children provided with an organic diet (Lu et al. 2006); and reduced urinary excretion of antibiotics and phthalates after a 5-day Buddhist “temple stay” that involved a vegetarian diet (Ji et al. 2010).

In the present study, we assessed changes in urinary BPA and phthalate metabolite levels during and after a 3-day dietary intervention designed to minimize exposure to food packaged in plastic or cans by substituting a “fresh-foods” diet. We measured phthalate metabolites that we expected to have substantial dietary sources and, for comparison, some metabolites for which diet is not expected to be a major source. We expected to see large reductions in BPA and the DEHP metabolites mono-2-ethylhexyl phthalate (MEHP), mono-(2-ethyl-5-oxohexyl) phthalate (MEOHP), and mono-(2-ethyl-5-hydroxyhexyl) phthalate (MEHHP). We expected smaller reductions in monobutyl phthalate (MBUP; a metabolite of DBP and BBP) and monobenzyl phthalate (MBZP; a metabolite of BBP) and little or no reduction in monoethyl phthalate (MEP; a metabolite of DEP) and monomethyl phthalate (MMEP; a metabolite of DMP).

## Materials and Methods

*Participants.* We selected five families to participate in a study to assess BPA and phthalate urine levels at three time periods: preintervention (while eating their typical diet); during intervention [on a special diet of fresh foods (no canned foods) prepared and packaged almost exclusively without contact with plastic]; and postintervention (after ending the special diet).

Sixty-three families in the greater San Francisco Bay Area of California responded to letters on five listservs by completing a brief online survey about demographic characteristics and diet over the previous 2 days [see Supplemental Material, Initial Recruitment Survey (doi:10.1289/ehp.1003170)]. To identify families whose diet included sources of BPA and phthalates, we asked families to complete a survey on certain dietary practices. Eligible families had two adults and two toilet-trained children 3–12 years of age, lived in the San Francisco Bay Area, had no significant dietary restrictions, and indicated either the consumption of canned foods or exposure to at least two of these potential sources of dietary BPA and phthalates: *a*) drank from personal water bottles, *b*) drank from large polycarbonate 2- to 5-gallon water bottles in office coolers, *c*) ate meals outside of the home, or *d*) ate meals microwaved in plastic. Of 63 families that completed the survey, 20 met the criteria for study inclusion. Three of these families could not participate because of logistical concerns (e.g., travel), and another three did not return calls. Based on telephone interviews with the remaining 14, we selected the 5 families who reported the most frequent consumption of canned foods and who seemed likely to be able to comply with the study protocol (e.g., we excluded potential participants who worked night shift, ate a low-carbohydrate diet). The age, family composition, and geographic location of the 9 nonparticipant families were similar to those of the 5 families who were enrolled. The Vassar College Institutional Review Board approved the study protocol.

*Dietary intervention.* A caterer, whom the research team had informed about possible sources of BPA and phthalates to avoid, developed an initial set of menu options. After reviewing these options and sharing them with participants to learn their preferences, the research team selected a final menu.

All families received the same foods for the 3-day meal intervention in January 2010. Intervention-period foods were prepared almost exclusively from fresh and organic fruits, vegetables, grains, and meats [see Supplemental Material, Table 1 (doi:10.1289/ehp.1003170)]. Preparation techniques avoided contact with plastic utensils and nonstick-coated cookware, and foods were stored in glass containers with BPA-free plastic lids. Containers were filled to below the top so foods did not contact the lids. Researchers instructed families to store foods only in these containers during the intervention and to avoid microwaving the lids. Participants received stainless steel water bottles and lunch containers to avoid other common sources of BPA and phthalates. Participants were encouraged to eat only the food provided during the intervention; they were advised that if they had to depart from the provided foods, they could use fresh foods, such as fruits, vegetables, eggs, peanut butter, and jelly from glass jars, and milk and orange juice from glass containers or low-density polyethylene plastic, if glass was not available. Coffee drinkers were advised to use a French press or ceramic drip rather than using a plastic coffee maker or buying coffee from a cafe.

**Table 1:** Characteristics of the 20 participants.

Table 1. Characteristics of the 20 participants.	
Characteristic	*n* (%)
Age (years)	
< 6	3 (15)
6 to < 12	7 (35)
12 to < 20	0 (0)
20 to < 40	4 (20)
40 to < 60	6 (30)
≥ 60	0 (0)
Ethnicity	
White	14 (70)
Hispanic	1 (5)
Asian	1 (5)
Mixed	4 (20)
Sex	
Male	9 (45)
Female	11 (55)
Urinary creatinine	
Preintervention	
< 118.6 mg/dL*a*	11 (55)
> 118.6 mg/dL	9 (45)
During intervention	
< 118.6 mg/dL	11 (55)
> 118.6 mg/dL	9 (45)
Postintervention	
< 118.6 mg/dL	13 (65)
> 118.6 mg/dL	7 (35)
**a**Creatinine data are classified as below or above the median (118.6 mg/dL) reported by Barr et al. (2005) for a 1988–1994 sample of 22,245 individuals (6–90 years of age).	

*Sample collection.* Before sample collection, all adult participants gave informed consent for themselves and their children. Families received prelabeled 125-mL amber-glass urine sampling containers (EP Scientific Products, Miami, OK, USA), a daily checklist of study activities, and guidelines for storing and heating foods during the intervention. The field director spoke with families daily to address questions and concerns and to remind the families of study requirements for the day. We recorded any reported deviations from the intervention diet at this time. Families also completed food questionnaires to characterize potential dietary sources of BPA and phthalates during the pre- and postintervention periods. Data collection spanned 8 consecutive days. On days 1 and 2, families ate their normal diet; on day 2, the researchers delivered food for days 3–5 prepared by a local caterer; and on days 6–8, families returned to preparing their own food.

**Figure 1:**
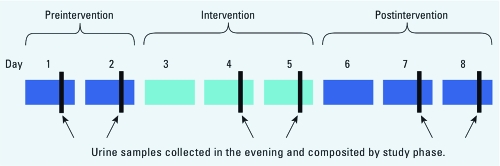
Intervention study design (*n* = 20 individuals from five families). Each participant provided a total of six urine samples (arrows; two per phase). Paired samples collected from each individual during each phase were combined for analysis.

Each participant provided a urine sample in the evening, usually after dinner, on days 1 and 2 (preintervention), 4 and 5 (intervention), and 7 and 8 (postintervention) (Figure 1). No samples were collected on days 3 and 6, while participants transitioned onto and off of the intervention. Families double-bagged urine specimen jars and stored them in their freezers until pickup within a week of the study’s conclusion. After pickup, urine samples were stored in a freezer overnight and shipped overnight on blue ice to the laboratory for processing and analysis. Samples were stored frozen at –20°C [for BPA < 2 weeks; for phthalate ester metabolite (PEM) < 8 weeks] before being thawed for analysis. After thawing, the laboratory archived aliquots of each individual urine sample at –20°C for possible future analysis.

*Laboratory analysis.* For each study phase (pre-, during, and postintervention) we combined the two urine samples collected from each individual. Both urine samples were thawed, and equal 40-mL volumes were combined in a clean 120-mL amber-glass jar. Once mixed, a 2-mL subsample was taken for creatinine measurement and 1-mL subsamples were taken for the BPA and PEM analytical  methods. Analysis was by HPLC/tandem mass spectrometry using isotope dilution quantification. See Supplemental Material (doi:10.​1289/ehp.1003170) for detailed extraction, analysis, and quantification methods.

Samples were analyzed in batches including quality control samples: a procedural blank, one spiked reference sample, and a reference sample in duplicate using laboratory stock urine for inter- and intrabatch comparisons. Most limits of detection (LODs) were around 1 ng/mL; LODs for MMEP and MEP were somewhat higher but mostly < 10 ng/mL. All quality control samples were within specifications for each batch. The laboratory was blinded to the identity of the samples, including which ones represented intervention or nonintervention collections.

*Data analysis.* Urinary concentrations are reported as analyte mass per volume (nanograms per milliliter), unadjusted for creatinine. Adjustment for creatinine is commonly used to reduce the impact of varying dilution on urinary biomarker concentrations. However, we addressed the influence of urine dilution by including creatinine as a variable in our model, as recommended by Barr et al. (2005), and we conducted confirmatory analyses using both unadjusted and creatinine-adjusted concentrations. These approaches were selected for a number of reasons. Creatinine concentrations have been shown to vary with protein content of the diet (Kesteloot and Joossens 1993; Neubert and Remer 1998) and therefore might be altered during the dietary intervention. Furthermore, because creatinine is associated with age and sex (Barr et al. 2005), adjusting for it might bias associations between urine metabolite concentrations and age or sex.

We calculated a method reporting limit (MRL) as the maximum of the sample-specific method LOD and the 90th percentile of the four laboratory blanks. We used all reported data, including measurements below the MRL. Twelve percent of MMEP measurements were reported as nondetects, and for these we assigned the sample-specific MRL (U.S. Environmental Protection Agency 2006). MRLs ranged from 0.25 ng/mL (BPA) to 7 ng/mL (MEP). Concentrations were not normally distributed but were approximately log-normal; therefore, we log-transformed concentrations for mixed-effects modeling and used nonparametric tests.

We used mixed-effects models for repeated measures, with family and participant included as multilevel random effects and creatinine as a fixed effect, to evaluate changes in concentrations over time. Specifically, we used a linear spline model with one knot placed at the middle time point, during the intervention. The impacts of age (adult/child as a categorical variable) and sex were evaluated as fixed effects. To corroborate our findings, we used Wilcoxon signed-rank tests on paired data to compare concentrations across two time periods (e.g., pre- and during intervention). Wilcoxon comparison of pre- and during intervention urine concentrations used both unadjusted and  creatinine-adjusted concentrations.

We evaluated the influence of being in the same family on exposure. To evaluate effects over the course of the study, we used variance estimates from the mixed-effects model. Specifically, we estimated the correlation among participants within the same family [the intraclass correlation (ICC)] as the variance attributable to the random effect of being in the same family divided by total variance (family, participant, and residual). We also estimated the percent variance explained by being in the same family by finding the difference in residual variance between models with and without family. In addition, to compare inter- and intrafamily variability at each time period, we used the nonparametric Kruskal-Wallis test, which evaluates the ratio of between- and within-group variability. Differences among families during the intervention are of particular interest because when diet is held constant, exposure variation due to other sources—some of which may be shared by families living together—can be observed.

We conducted data management and analysis in R (version 12.11.0; R Development Core Team 2010). All statistical tests were conducted at the 0.05 significance level.

## Results

Twenty participants (four members in each of five families) completed the dietary intervention study and provided a total of six urine samples (two samples collected during each phase of the study). We later combined these to make one sample per phase for each participant (Figure 1). The median age of the 10 adults was 40.5 years, and the median age of the 10 children was 7 years. Characteristics of study participants are provided in Table 1.

**Figure 2:**
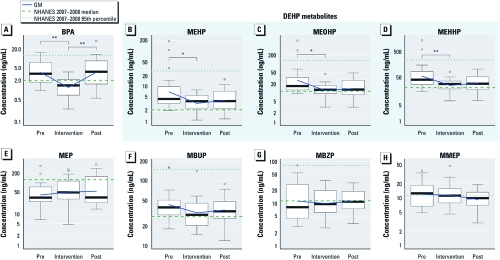
Box plots showing the distribution of urinary levels of BPA and phthalate metabolites in preintervention (Pre), intervention, and postintervention (Post) samples. Boxes represent values between the 25th and 75th percentiles; black lines inside boxes indicate medians; whiskers indicate the range of nonoutlier data points (using Tukey’s definition of outliers); and circles represent outliers. The fresh food intervention was associated with significant reductions in urinary excretion of BPA (*A*) and metabolites of DEHP [MEHP (*B*), MOHP (*C*), and MEHHP (*D*)]. No significant changes were observed in the other phthalate metabolites analyzed, although there was a small reduction in the DBP metabolite MBUP (*F*); concentrations of MEP (*E*), MBZP (*G*), and MMEP (*H*) showed little change. Compared with the 2007–2008 NHANES sample of 2,604 individuals ≥ 6 years of age (CDC 2009), the preintervention medians and 95th percentile estimates for adults and children combined were higher for BPA (*A*), DEHP metabolites (*B*–*D*), MBUP (*F*), and MMEP (*H*), much lower for MEP (*E*), and similar for MBZP (*G*). The NHANES median for MMEP was < LOD of 1.1 ng/mL, and the NHANES 95th percentile for MEP was 2,140 ng/mL. *p < 0.05, and **p < 0.005 for reductions or increases between intervention phases as determined by *p*-value for slope in the mixed-effects model.

We detected all but one of the analytes in 100% of the samples; MMEP was detected in 88% of samples. Compared with the 2007–2008 National Health and Nutrition Examination Survey (NHANES) sample of 2,604 individuals ≥ 6 years of age [Centers for Disease Control and Prevention (CDC) 2009], preintervention medians and 95th percentile estimates for adults and children combined in the present study were higher for BPA and metabolites of DEHP (MEHP, MEHHP, and MEOHP) and for MBUP (a metabolite of DBP and BBP) and MMEP (a metabolite of DMP); much lower for the DEP metabolite MEP; and similar for the BBP metabolite MBZP (Figure 2, Table 2). Higher overall median values for BPA and DEHP were due to higher median values in adult study participants than in NHANES adults, whereas children’s levels were similar to NHANES median values for children. The preintervention creatinine medians were similar to those derived from the 1988–1994 NHANES sample of 22,245 individuals (Barr et al. 2005).

**Table 2:** Preintervention concentrations of urinary analytes.

Table 2. Preintervention concentrations of urinary analytes.																						
				Adults (*n* = 10)						NHANES adult median*a*		Children (*n* = 10)						NHANES child median*b*		Study combined median		NHANES overall median*c*
Analyte		MRL		Min		Median		Max				Min		Median		Max						
Creatinine (mg/dL)				58		150		220		119–128.8		33		68		160		98.09		100		118.6
BPA (ng/mL)		0.25		1.0		4.9		11		2		1.2		2.6		16		2.4		3.4		2.1
MEHP (ng/mL)		1		3.3		7.4		190		2.1		2.1		3.9		15		2.2		4.5		2.2
MEOHP (ng/mL)		1		9.5		24		630		10.7		11		16		66		16.5		17		11.4
MEHHP (ng/mL)		1		22		50		1,400		19.6		15		35		150		27		42		20.7
MEP (ng/mL)		7		32		73		340		128		6.7		25		39		68.7		34		124
MBUP*d* (ng/mL)		1		18		30		160		26		34		46		160		40.1		39		28
MBZP (ng/mL)		1		2.9		8.0		32		9.9		3.9		12		82		24.2		8.3		11.7
MMEP (ng/mL)		5		< MRL*e*		8.1		34		< 1.1		5.7		15		38		1.2		13		< 1.1
Abbreviations: Max, maximum; Min, minimum. **a**Data include creatinine medians for 30- to 39-year-olds and 40- to 49-year-olds from the Barr et al. (2005) analysis of 1988–1994 NHANES data (*n* = 3,259 and 2,542), and BPA and phthalate medians for ≥ 20 years of age from 2007–2008 data (CDC 2009; *n* = 1,814). **b**Data include creatinine medians for 6- to 11-year-olds from Barr et al. (2005; *n* = 3,078) and BPA and phthalate medians for 6- to 11-year-olds from 2007–2008 data (CDC 2009; *n* = 389). **c**Data include creatinine medians for 6- to 90-year olds from Barr et al. (2005; *n* = 22,245) and BPA and phthalate medians for 6- to ≥ 85-year-olds from 2007–2008 data (CDC 2009; *n* = 2,604). **d**We did not distinguish between mono-*n*-butyl phthalate and monoisobutyl phthalate in the present study; therefore, NHANES medians presented for MBUP are the sum of these two forms. **e**Three samples were < MRL for MMEP, and one could not be analyzed for MMEP.																						

Urinary geometric mean (GM) values of BPA and of the DEHP metabolites MEHP, MEHHP, and MEOHP were significantly lower during the intervention than before the intervention (Figure 2, Table 3). GMs were reduced 66%, 53%, 55%, and 56% for BPA, MEHP, MEOHP, and MEHHP, respectively. We observed similar findings with the paired Wilcoxon signed-rank tests for unadjusted and creatinine-adjusted concentrations for BPA and the three DEHP metabolites [see Supplemental Material, Figures 1 and 2 (doi:10.1289/ehp.1003170)], although the decrease was not statistically significant for creatinine-adjusted MEHP and MEOHP. Reductions in the upper ends of the exposure distributions were larger than corresponding reductions in the GM values (Figure 2; see also Supplemental Material, Table 2). For example, the 90th percentiles of BPA and MEHP were reduced by 73% and 84%, respectively, and maxima were reduced by 76% and 96%. Consistent with the greater reduction at the tops of the exposure distributions, the lower GMs during the intervention were accompanied by smaller interquartile ranges (reductions of 75%, 48%, 64%, and 68% for BPA, MEHP, MEOHP, and MEHHP, respectively) (see Supplemental Material, Table 2). Among the phthalates other than DEHP metabolites, we observed a nonsignificant 25% reduction in MBUP and no clear differences for other analytes (Table 3).

**Table 3:** Mixed-effects model results for multilevel spline model.

Table 3. Mixed-effects model results for multilevel spline model.						
Analyte		Intervention variable		Percent change in GM per time period (ng/mL)*a *		95% CI for slope estimate
BPA		Pre vs. during*b *		–66% (3.7 vs. 1.2)		–1.6 to –0.55**
		During vs. post		202% (1.2 vs. 3.8)		0.61 to 1.6**
MEHP		Pre vs. during		–53% (7.1 vs. 3.4)		–1.2 to –0.16*
		During vs. post		21% (3.4 vs. 4.1)		–0.32 to 0.74
MEOHP		Pre vs. during		–55% (27 vs. 12)		–1.2 to –0.2*
		During vs. post		16% (12 vs. 14)		–0.35 to 0.69
MEHHP		Pre vs. during		–56% (57 vs. 25)		–1.3 to –0.25*
		During vs. post		22% (25 vs. 31)		–0.3 to 0.72
MEP		Pre vs. during		23% (41 vs. 50)		–0.059 to 0.7
		During vs. post		7% (50 vs. 53)		–0.28 to 0.47
MBUP		Pre vs. during		–25% (43 vs. 32)		–0.44 to 0.043
		During vs. post		11% (32 vs. 35)		–0.12 to 0.36
MBZP		Pre vs. during		–12% (12 vs. 10)		–0.38 to 0.36
		During vs. post		13% (10 vs. 11)		–0.22 to 0.51
MMEP		Pre vs. during		–4% (12 vs. 12)		–0.28 to 0.32
		During vs. post		–19% (12 vs. 9.3)		–0.5 to 0.091
Abbreviations: CI, confidence interval; Post, postintervention; Pre, Preintervention. **a**Percent change in the GM between the two time periods, with GMs of the two time periods shown in parentheses. **b**Each change estimate represents the slope between the two time periods. *p < 0.05, and **p < 0.005. The intercept (which represents the log concentration during the intervention) was significant for all models except BPA. Creatinine and age (adult vs. child) were included in all models; creatinine was significant for MEOHP, MEP, MBUP, MBZP, MMEP; age was significant for MEP, MBUP, MBZP, and MMEP; sex was not significant in any of the models and was not included in the final models.						

After participants returned to their regular diets, BPA levels increased to approximately preintervention levels (*p* < 0.01) (Figure 2, Table 3). We also observed a significant increase in BPA in paired Wilcoxon signed-rank tests using adjusted and unadjusted concentrations [see Supplemental Material, Figures 1 and 2 (doi:10.1289/ehp.1003170)]. The GMs of DEHP metabolites increased by 16–22% after the intervention, although this change was not statistically significant (Figure 2, Table 3). Creatinine concentrations were reduced by the intervention (GMs, 94 mg/dL vs. 76 mg/dL; paired Wilcoxon signed-rank test, *p* = 0.04).

Urinary concentrations did not differ significantly between adults and children for BPA, but some differences were observed for phthalate metabolites. In the mixed-effects model, we observed significant differences in urinary concentrations between adults and children across the study period for MEP, MBUP, MBZP, and MMEP (*p* < 0.05; data not shown). Adults had significantly higher concentrations than did children for MEP (GMs for adults vs. children: preintervention, 78 vs. 21 ng/mL; during intervention, 92 vs. 27 ng/mL; postintervention, 98 vs. 29 ng/mL),  whereas children had significantly higher concentrations of MBUP (GMs for adults vs. children: preintervention, 34 vs. 53 ng/mL;  during intervention, 35 vs. 29 ng/mL; postintervention, 32 vs. 38 ng/mL), MBZP (preintervention, 9.3 vs. 14 ng/mL; during intervention, 11 vs. 9.3 ng/mL; postintervention, 8.3 vs. 16 ng/mL), and MMEP [preintervention, 11 vs. 13 ng/mL;  during intervention, 10 vs. 13 ng/mL; post intervention, 8.7 vs. 10 ng/mL) (see Supplemental Material, Table 2 (doi:10.1289/ehp.1003170)]. Males and females did not differ significantly; therefore, sex was not included in the final model (data not shown).

*Effects of family membership on exposure.* We used variance estimates from the mixed- effects model to estimate the correlations among participants within the same family (ICC) and the percentage of total variance explained by inclusion of family as a random effect. The estimated ICCs range from approximately zero for MEP to 0.27 for MMEP, indicating substantial variation within families (data not shown). The only analyte with a substantial percent variance explained by family membership was BPA (11%). In contrast, the percent variance explained by family membership was only 3.6% for MMEP, 2.6% for MBUP, and near zero for other metabolites.

Variation in urinary BPA was greater among families than within families during the intervention (Kruskal-Wallis test, *p* < 0.01) but not at any other time. Urinary concentrations during the intervention of individuals grouped by family are shown in Supplemental Material, Figure 3 (doi:10.1289/ehp.1003170). We observed significant variation in MEOHP and MEHHP among families after the intervention (data not shown) and variation in MMEP among families during and after the intervention (Kruskal-Wallis test, *p* < 0.05).

*Dietary sources.* Based on daily contacts with the research staff, participant-reported compliance with the intervention protocol was high, and the few substitutions reported by participants were within the options specified in the instructions. Reported deviations from the intervention diet are presented in Supplemental Material, Table 3 (doi:10.1289/ehp.1003170).

Potential exposure sources recorded during the 2 days before and 3 days after the intervention included meals prepared outside the home, canned foods, canned soda, frozen dinners, drinking from polycarbonate water bottles, and microwaving in plastic. All families reported using canned foods or having at least one meal outside the home during the pre- or postintervention phases of the study. Two families reported microwaving frozen meals in plastic. Seven of 10 adults and 5 of 10 children had canned soda. One participant reported repeated use of a polycarbonate beverage container, and one family reported drinking from a multigallon polycarbonate drinking water container on one occasion. The self-reported diet data we collected [see Supplemental Material, Table 4 (doi:10.1289/ehp.1003170)] were too limited to support statistical analysis of dietary predictors of high BPA and PEM levels.

## Discussion

In this study, GM urinary BPA concentrations fell by 66% and GM DEHP metabolite concentrations fell by 53–56% when participants began a “fresh foods” diet, suggesting that most BPA and DEHP intake came from food packaging or meals outside the home. Maxima declined 76% for BPA and 93–96% for DEHP metabolites, showing a dramatic reduction in the range of exposures while participants were eating fresh foods. In contrast, the DBP metabolite MBUP decreased nonsignificantly, and other phthalates showed little or no effect.

Participants’ reports of their food practices suggested that canned foods and beverages and restaurant meals were the most likely sources of exposure to BPA and DEHP in their usual diets, because participants reported limited use of polycarbonate water bottles, frozen prepared foods, and microwaving in plastic. This inference is consistent with NHANES data showing higher BPA levels associated with consumption of meals prepared out of the home, sodas, and school lunches (canned foods were not assessed) (Lakind and Naiman 2010). Exposure to PVC film, commonly used in food storage at home and in restaurants, may be another important exposure source, because these films are known to contain BPA and DEHP (Lopez-Cervantes and Paseiro-Losada 2003; Petersen and Jensen 2010) and were not used during the intervention in the present study. Our intervention limited exposures to canned foods and plastic food packaging by substituting fresh foods prepared from basic ingredients; however, it is difficult to determine exactly which of these changes in food sourcing and handling were responsible for the significant exposure reductions we observed.

Our findings are consistent with estimates that predict dietary intake as a major source of BPA and DEHP exposure (NTP-CERHR 2006; Willhite et al. 2008; Wormuth et al. 2006). Although DBP and BBP exposures are also predicted to be substantially from diet, we observed relatively little or no change in their metabolites (MBUP and MBZP). For DEP, diet is not expected to be a major source, and we saw no reduction in its metabolite MEP.

Our intervention did not eliminate all dietary sources of exposure. Food contamination may occur during premarket processing of whole foods or from the presence of phthalates and BPA in the environment from which the food originates. One example is the migration of DEHP into milk from PVC tubing used in the milking process (Feng et al. 2005). In addition, BPA, DBP, and DEHP have been detected in whole eggs sold in Asia, demonstrating the possibility for contamination before preparation and packaging (e.g., Shao et al. 2007). Thus, we are not surprised that exposure reductions in the present study were not as large as predicted from the NTP exposure assessments for BPA and DEHP, which estimated diet as the source of 99% and 90% of exposure, respectively (NTP-CERHR 2006, 2008). Our findings of little or no influence of the intervention on DBP and BBP could mean that these compounds enter food upstream of our intervention; that packaging formulations have changed between the NTP estimates and our 2010 intervention; or that nondietary sources are a larger component of exposure than predicted. The report by Colacino (2010) showing that NHANES participants with higher vegetable intake had higher urinary MEP suggests the possibility that the nonsignificant increase in DEP during our intervention was due to higher vegetable intake, or our finding could be due to chance.

*Implications of chemical differences in clearance time.* Although BPA levels increased between the intervention and postintervention samples, returning to preintervention levels, concentrations of DEHP metabolites increased only slightly. This could reflect a longer clearance time for DEHP than for BPA. Estimates of elimination half-lives in primates or humans are 3–6 hr for BPA (Doerge et al. 2010; Taylor et al. 2011; Willhite et al. 2008) and 15–24 hr for DEHP (Koch et al. 2005). Thus, the 1-day lags between sampling periods may have been well suited for BPA pharmacokinetics but too short to fully capture changes in DEHP intake. This problem is more likely to affect the postintervention “rebound” because the effective clearance time was shorter. The time lag from preintervention to intervention samples (when DEHP metabolites decreased) was 48 hr (from dinner on day 2 to the first collection of intervention urine on day 4), whereas the lag from intervention to postintervention was effectively about 34 hr (probably from breakfast or lunch on day 6 to the first collection of postintervention urine on day 7). This approximately 14-hr discrepancy may explain the absence of noticeable rebound effects in the levels of DEHP metabolites. This explanation is also supported by the nonsignificant increase in DEHP metabolite levels between intervention and postintervention collections, and by the nonsignificant decrease in postintervention levels compared with preintervention. Alternatively, persistent (after the intervention) participant changes in behaviors that affect DEHP but not BPA exposure could potentially explain the pattern of DEHP levels after the intervention.

*Residual shared family exposures.* In general, membership in a family did not have a large effect on exposure over the entire study period. However, during the intervention, when many dietary sources were controlled, we found significant between-family variation for BPA, suggesting that other key exposures are shared within a family. These shared exposures likely occur in the home and may be due to direct contact with BPA-containing materials or exposure to BPA in house dust or indoor air (Rudel et al. 2003, 2010). For the phthalates (except MMEP), we did not observe significant between-family variation during the intervention, suggesting that individual behaviors are relatively more important than the shared home environment.

*Differences between adults and children and by sex.* Mixed-effects models and Wilcoxon tests indicated significantly higher levels of the DEP metabolite MEP in adults than in children. Mixed-effects models also indicated significantly higher levels of the other phthalate metabolites (MBUP, MBZP, and MMEP) in children compared with adults across the study period. The difference between DEP and other phthalates may originate from differences in intake rate or exposure sources. We observed no significant differences between males and females.

*Limitations.* Although effect estimates were statistically significant using multiple approaches, our sample size was small, and we cannot rule out the possible role of chance in our findings. However, an intervention study—where individuals serve as their own controls—avoids many sources of variation that can confound findings in cross-sectional studies.

Generalizability from this sample to the U.S. population is limited because the relatively small number of participants in a particular geographic location may not be representative. In addition, we intentionally selected participants who reported consuming packaged and prepared foods expected to contain BPA and DEHP. If these participants consumed more packaged and prepared foods than typical Americans, our results could overstate the role of these sources in overall exposure. However, our observation that exposures before the intervention were generally in the range of those reported for the U.S. population by the CDC (Calafat et al. 2008) suggests that our findings are likely to be broadly relevant to American diets.

Although participants reported high compliance with the study intervention, we cannot be sure that all deviations from the intervention diet were reported. The consumption of nonapproved foods during the intervention might have reduced the effect of the intervention. In addition, families may have responded to study information by lowering their intake of BPA- or phthalate-containing foods at any time before or during the study, reducing the effect of the intervention.

## Conclusions

Three days of eating food with limited food packaging was associated with substantial reductions in BPA and DEHP exposures. Results of this study suggest that removing BPA and DEHP from food packaging will significantly decrease exposure for adults and children. More generally, these results illustrate how intervention studies of chemicals in consumer products can inform regulatory decision making, product formulation, and consumer choices.

## Supplemental Material

(232 KB) PDF

## References

[r1] Barr DB, Wilder LC, Caudill SP, Gonzalez AJ, Needham LL, Pirkle JL (2005). Urinary creatinine concentrations in the U.S. population: implications for urinary biologic monitoring measurements.. Environ Health Perspect.

[r2] Calafat AM, Ye X, Wong LY, Reidy JA, Needham LL (2008). Exposure of the U.S. population to bisphenol A and 4-*tertiary*-octylphenol: 2003–2004.. Environ Health Perspect.

[r3] Carwile JL, Luu HT, Bassett LS, Driscoll DA, Yuan C, Chang JY (2009). Polycarbonate bottle use and urinary bisphenol A concentrations.. Environ Health Perspect.

[r4] CDC (Centers for Disease Control and Prevention) (2009). Fourth National Report on Human Exposure to Environmental Chemicals.. http://www.cdc.gov/exposurereport/data_tables/index.html#DataTablesByChemicalGroup.

[r5] Colacino JA, Harris TR, Schecter A (2010). Dietary intake is associated with phthalate body burden in a nationally representative sample.. Environ Health Perspect.

[r6] Doerge DR, Twaddle NC, Woodling KA, Fisher JW (2010). Pharmacokinetics of bisphenol A in neonatal and adult rhesus monkeys.. Toxicol Appl Pharmacol.

[r7] Engel SM, Zhu C, Berkowitz GS, Calafat AM, Silva MJ, Miodovnik A (2009). Prenatal phthalate exposure and performance on the Neonatal Behavioral Assessment Scale in a multiethnic birth cohort.. Neurotoxicology.

[r8] European Food Safety Authority (2005). Opinion of the Scientific Panel on Food Additives, Flavourings, Processing Aids and Materials in Contact with Food (AFC) on a request from the Commission related to bis (2-ethylhexyl) phthalate (DEHP) for use in food contact materials. EFSA J 243:1–20.. http://www.efsa.europa.eu/en/efsajournal/doc/243.pdf.

[r9] FDA (Food and Drug Administration) (2010). Update on Bisphenol A for Use in Food Contact Applications: January 2010.. http://www.fda.gov/NewsEvents/PublicHealthFocus/ucm197739.htm.

[r10] Feng YL, Zhu JP, Sensenstein R (2005). Development of a headspace solid-phase microextraction method combined with gas chromatography mass spectrometry for the determination of phthalate esters in cow milk.. Anal Chim Acta.

[r11] Food and Agriculture Organization of the United Nations/World Health Organization (2010). Joint FAO/WHO Expert Meeting to Review Toxicological and Health Aspects of Bisphenol A: Summary Report.. http://www.who.int/entity/foodsafety/chem/chemicals/BPA_Summary2010.pdf.

[r12] Fromme H, Gruber L, Schlummer M, Wolz G, Bohmer S, Angerer J (2007). Intake of phthalates and di(2-ethylhexyl)adipate: results of the Integrated Exposure Assessment Survey based on duplicate diet samples and biomonitoring data.. Environ Int.

[r13] Gray LE, Ostby J, Furr J, Price M, Veeramachaneni DN, Parks L (2000). Perinatal exposure to the phthalates DEHP, BBP, and DINP, but not DEP, DMP, or DOTP, alters sexual differentiation of the male rat.. Toxicol Sci.

[r14] Hauser R. (2008). Urinary phthalate metabolites and semen quality: a review of a potential biomarker of susceptibility.. Int J Androl.

[r15] Itoh H, Yoshida K, Masunaga S. (2007). Quantitative identification of unknown exposure pathways of phthalates based on measuring their metabolites in human urine.. Environ Sci Technol.

[r16] Ji K, Lim Kho Y, Park Y, Choi K. (2010). Influence of a five-day vegetarian diet on urinary levels of antibiotics and phthalate metabolites: a pilot study with “temple stay” participants.. Environ Res.

[r17] Kesteloot HE, Joossens JV (1993). Relationship between dietary protein intake and serum urea, uric acid and creatinine, and 24-hour urinary creatinine excretion: the BIRNH Study.. J Am Coll Nutr.

[r18] Koch HM, Bolt HM, Preuss R, Angerer J (2005). New metabolites of di(2-ethylhexyl)phthalate (DEHP) in human urine and serum after single oral doses of deuterium-labelled DEHP.. Arch Toxicol.

[r19] Lakind JS Naiman DQ 2010 Daily intake of bisphenol A and potential sources of exposure: 2005–2006 National Health and Nutrition Examination Survey. J Expo Sci Environ Epidemiol 21 272 279doi:[Online 17 March 2010] 10.1038/jes.2010.9 20237498 PMC3079892

[r20] Lopez-Cervantes J, Paseiro-Losada P. (2003). Determination of bisphenol A in, and its migration from, PVC stretch film used for food packaging.. Food Addit Contam.

[r21] Lu C, Toepel K, Irish R, Fenske RA, Barr DB, Bravo R (2006). Organic diets significantly lower children’s dietary exposure to organophosphorus pesticides.. Environ Health Perspect.

[r22] Meeker JD, Calafat AM, Hauser R (2007). Di(2-ethylhexyl) phthalate metabolites may alter thyroid hormone levels in men.. Environ Health Perspect.

[r23] Meeker JD, Calafat AM, Hauser R (2009). Urinary metabolites of di(2-ethylhexyl) phthalate are associated with decreased steroid hormone levels in adult men.. J Androl.

[r24] Neubert A, Remer T. (1998). The impact of dietary protein intake on urinary creatinine excretion in a healthy pediatric population.. J Pediatr.

[r25] NTP-CERHR (National Toxicology Program Center for the Evaluation of Risks to Human Reproduction) (2003a). NTP-CERHR Monograph on the Potential Human Reproductive and Developmental Effects of Butyl Benzyl Phthalate (BBP). NIH 03-4487.. http://ntp.niehs.nih.gov/ntp/ohat/phthalates/bb-phthalate/BBP_Monograph_Final.pdf.

[r26] NTP-CERHR (National Toxicology Program Center for the Evaluation of Risks to Human Reproduction) (2003b). NTP-CERHR Monograph on the Potential Human Reproductive and Developmental Effects of Di-*n*-Butyl butyl Phthalate (DBP).. http://ntp.niehs.nih.gov/ntp/ohat/phthalates/dbp/DBP_Monograph_Final.pdf.

[r27] NTP-CERHR (National Toxicology Program Center for the Evaluation of Risks to Human Reproduction) (2006). NTP-CERHR Monograph on the Potential Human Reproductive and Developmental Effects of Di(2-Ethylhexyl) Phthalate. NIH 06-4476.. http://ntp.niehs.nih.gov/ntp/ohat/phthalates/dehp/DEHP-Monograph.pdf.

[r28] NTP-CERHR (National Toxicology Program Center for the Evaluation of Risks to Human Reproduction) (2008). NTP-CERHR Monograph on the Potential Human Reproductive and Developmental Effects of Bisphenol A. NIH 08-5994.. http://ntp.niehs.nih.gov/ntp/ohat/bisphenol/bisphenol.pdf.

[r29] Petersen JH, Jensen LK (2010). Phthalates and food-contact materials: enforcing the 2008 European Union plastics legislation.. Food Addit Contam Part A Chem Anal Control Expo Risk Assess.

[r30] R Development Core Team (2010). R: A Language and Environment for Statistical Computing..

[r31] Rudel RA, Camann DE, Spengler JD, Korn LR, Brody JG (2003). Phthalates, alkylphenols, pesticides, polybrominated diphenyl ethers, and other endocrine-disrupting compounds in indoor air and dust.. Environ Sci Technol.

[r32] Rudel RA, Dodson RE, Perovich LJ, Morello-Frosch R, Camann DE, Zuniga MM (2010). Semivolatile endocrine-disrupting compounds in paired indoor and outdoor air in two northern California communities.. Environ Sci Technol.

[r33] Schecter A Malik N Haffner D Smith S Harris TR Paepke O 2010 Bisphenol A (BPA) in U.S. Food. Environ Sci Technol 44 9425 9430doi:[Online 1 November 2010] 10.1021/es102785d 21038926

[r34] Shao B, Han H, Tu X, Huang L. (2007). Analysis of alkylphenol and bisphenol A in eggs and milk by matrix solid phase dispersion extraction and liquid chromatography with tandem mass spectrometry.. J Chromatogr B.

[r35] Swan SH (2008). Environmental phthalate exposure in relation to reproductive outcomes and other health endpoints in humans.. Environ Res.

[r36] Taylor JA, Vom Saal FS, Welshons WV, Drury B, Rottinghaus G, Hunt PA (2011). Similarity of bisphenol A pharmacokinetics in rhesus monkeys and mice: relevance for human exposure.. Environ Health Perspect.

[r37] U.S. Environmental Protection Agency (2006). Data Quality Assessment: Statistical Methods for Practitioners. EPA QA/G-9S.. http://www.epa.gov/QUALITY/qs-docs/g9s-final.pdf.

[r38] Willhite CC, Ball GL, McLellan CJ (2008). Derivation of a bisphenol A oral reference dose (RfD) and drinking-water equivalent concentration.. J Toxicol Environ Health B Crit Rev.

[r39] Wormuth M, Scheringer M, Vollenweider M, Hungerbuhler K. (2006). What are the sources of exposure to eight frequently used phthalic acid esters in Europeans?. Risk Anal.

