# Supplementary material for: Food Packaging and Bisphenol A and Bis(2-Ethyhexyl) Phthalate Exposure: Findings from a Dietary Intervention
Source: Environ Health Perspect. 2011 Mar 30;119(7):914–20. doi: 10.1289/ehp.1003170 (PMC3223004; doi:10.1289/ehp.1003170)
Supplement: (232 KB) PDF [file ehp.1003170.s001.pdf]

Supplemental Material for Environmental Health Perspectives

Food Packaging and Bisphenol A and Bis(2-Ethyhexyl) Phthalate Exposure: Findings from a Dietary Intervention

Ruthann A. Rudel,[1]* Janet M Gray,[2,3] Connie L. Engel,[2] Teresa W. Rawsthorne,[4] Robin E. Dodson,[1] Janet M. Ackerman,[1] Jeanne Rizzo,[2] Janet L. Nudelman,[2] Julia Green Brody[1]

[1]Silent Spring Institute, Newton, MA 02458
[2]Breast Cancer Fund, San Francisco, CA 94109
[3]Vassar College, Poughkeepsie, NY 12604
[4]AXYS Analytical Services, Sidney, British Columbia V8L 5X2, Canada

*Address correspondence to Ruthann Rudel, Silent Spring Institute, 29 Crafts Street, Newton, MA 02458; phone 617-332-4288 x214; fax 617-332-4284; rudel@silentspring.org

Supplemental Material:

- Detailed Analytical Methods (page 2)
- Initial Recruitment Survey (page 4)
- Supplemental Material Table 1, Intervention diet (page 8)
- Supplemental Material Table 2, Urinary BPA and phthalate levels pre-intervention, during intervention and post-intervention for (a) all subjects, (b) children, and (c) adults (page 9)
- Supplemental Material Table 3, Self-reported dietary deviations during intervention (page 12)
- Supplemental Material Table 4, Possible sources of BPA and phthalates before and after dietary intervention (page 13)
- Supplemental Material Figure 1, Boxplots of unadjusted urinary concentrations with Wilcoxon signed rank test results (page 14)
- Supplemental Material Figure 2, Boxplots of creatinine-adjusted urinary concentrations with Wilcoxon signed rank test results (page 15)
- Supplemental Material Figure 3, Urinary levels of each analyte during dietary intervention, shown by family (page 16)

Detailed Analytical Methods

To composite samples, urine was thawed and two equal 40 mL volumes were combined in a clean 120 mL amber glass jar. Once mixed, a 2 mL subsample was taken for creatinine measurement and 1 mL subsamples were taken for the BPA and PEM analytical methods. Analysis was by HPLC/MS/MS using isotope dilution quantification.

For the subsamples taken for BPA and PEM analysis, ammonium acetate buffer solution was added to 1 mL of the urine sample, followed by the addition of a isotopically labeled surrogate standard solution (d6-BPA or 13C-labeled PEMs, Cambridge Isotope Laboratories) and an aliquot of 4-methyl belliferyl glucuronide (used to monitor the completeness of the enzymatic hydrolysis). To the sample mixture was then added β-glucuronidase enzyme solution followed by incubation at 37°C for 3 hours. Following dilution of the sample mixture with water and adjustment to PH2, the samples were extracted and cleaned up using Waters Oasis glass HLB 200 mg, 5 mL, solid-phase extraction glass cartridges. The extracts were then spiked with labeled recovery standards (13C12-BPA or 13C4 mono-n-octyl phthalate, CIL) to calculate internal standard recovery.

Analysis of sample extracts for phthalates and BPA used a Waters 2695 HPLC coupled with a triple quadrapole mass spectrometer (Micromass Quattro Ultima MS/MS, LC column PEMs: Sunfire C18 3.5 um, 46 mm X 30mm analytical column; LC column BPA: Waters Xterra C18MS, 10 cm, 2.1 mm id, 3.5 um analytical column). Separate HPLC-MSMS runs in the electrospray negative ion mode were conducted, one for the phthalate metabolites and one for BPA. The HPLC/MS/MS for each target analyte was run at unit mass resolution in the Multiple Reaction Monitoring mode and resulting measurements were produced by the manufacturer's Mass Lynx v.4.0 software.

Samples were analyzed in batches including quality control samples: a procedural blank, one spiked reference sample, and a reference sample in duplicate using laboratory stock urine for inter- and intra- batch comparisons. On the basis of spiked recovery standards, a ‘‘specimen detection limit’’ was determined for each sample by converting the area equivalents corresponding to three times the height of the chromatographic noise to a concentration (in the same way that peak areas are converted to concentrations). The method detection limit of each assay was calculated as the greater of two concentrations: (1) the lowest calibration standard converted to a sample equivalent concentration or (2) the sample-specific detection limit. All analyte detection limits were ≤ 1 ng/mL, except MMeP in one sample had a 2.15 ng/mL detection limit. All quality control samples were within specifications for each batch. The laboratory was blind to the identity of the samples, including which ones represented intervention or non-intervention collections.

1. Default Section

1. Please list the ages and genders of your immediate family who live at home with you. Include yourself.

	Age	Gender
You		
Other Parent		
Child 1		
Child 2		

2. Where do you live?

City	
County	
ZIP Code	

3. Are you willing to do the following?

	Yes	No	Not Sure
If selected, my family is willing to eat catered meals made with fresh ingredients for all meals and snacks for three consecutive days (Tues-Thurs). Meals can be packed to go.			
If selected, my family is willing to provide six urine samples from each family member over the course of a week.			
In order to provide meals and collect urine samples, members of our research team will visit your home most nights for a full week, typically between 6pm and 8pm. Visits will be brief, and you will meet our team in advance. Can at least one adult family member be home each evening of the study week to meet with a member of our team?			
As far as you know at this point, will you be available to participate in this study from January 24-31?			

4. Please list prescription medications taken by each family member. Use the same entry order as question 1.

Me:	
Other Parent:	
Child 1:	
Child 2:	

As the main provider of food and meals to your family, please describe the foods you and your family have eaten over the past two full days before today (yesterday and the day before yesterday). You may want to stop and think for a few minutes about what you have prepared and eaten. Sometimes it's hard to remember right away what we have eaten in the past couple of days, but with a few minutes of thinking about it, we hope you can recall what you and your family ate recently.

What did your family eat in the last two days? Please list.

5. Breakfast yesterday

6. Breakfast, the day before yesterday

7. Lunch Yesterday

8. Lunch, the day before yesterday

9. Dinner Yesterday

10. Dinner, the day before yesterday

11. Snacks Yesterday

12. Snacks, the day before yesterday

13. Did you prepare the foods you ate at home or were they prepared somewhere else (restaurant, cafeteria, deli, prepared frozen food)? Check all that apply

	Prepared at Home	Prepared at a Restaurant	Prepared at a Cafeteria	From a deli or other place providing fresh (non-frozen) food	Frozen food
Breakfasts					
Lunches					
Dinners					
Snacks					
If your child or partner ate different food from you, to the best of their knowledge, was their food:					

14. When you prepared foods at home, how did you heat them? Check all that apply.

- On the Stove
- In the Oven/toaster oven
- In a microwave

15. If you heated your food in a microwave, was the food in: (Check all that apply to the foods you prepared in the past two days)

- The container you bought it in
- A plastic food storage container or bowl
- A glass or ceramic food storage container or bowl
- A paper plate or bowl
- A foam plate or bowl

Other (please specify)

16. When you or your family drank water, where did your water come from? (Check all that apply)

- Tap (faucet - filtered or unfiltered)
- Plastic filter pitcher
- Individual water bottle
- Gallon water bottle
- Larger (2-5 gallon water container, often found in offices or schools)

Other (please specify)

17. In preparing meals and snacks over the past two days, did you use? (Check all that apply)

- Fresh fruits and vegetables
- Frozen vegetables prepared in microwaveable pouches
- Frozen vegetables that you heated in your own container
- Canned fruits and vegetables

Other (please specify)

Done

Supplemental Material, Table 1. Intervention diet

Foods provided	Comments
**Breakfasts**	
Bran and blueberry muffins[a]	Made with fresh blueberries
Cinnamon rolls[a]	
Granola[a]	
Fresh fruit mix[a]	
Yogurt	From polypropylene container
**Lunches**	
Turkey	
Stone-ground mustard[a]	
Swiss cheese	
Tuna salad	Tuna from retort packaging - LDPE liner
Chicken taco filling[a]	
Vegetable bow tie pasta[a]	
Vegetable sticks[a]	Fresh carrots, celery
Fresh fruit[a]	Apples, bananas
Toasted nuts[a]	From bulk food bins
Bread	Organic, from grocery store
Flour tortillas	
**Dinners**	
Chicken and dumplings[a]	
Salad[a]	Organic, seasonal greens
Salad dressing[a]	Made with oils, vinegars in glass bottles
Crispy oven-fried chicken[a]	
Mashed potatoes[a]	
Sauteed spinach[a]	Fresh spinach
Deep-dish lasagna[a]	Fresh vegetables for filling and sauce
**Snacks**	
Apple crisp[a]	With fresh apples
Brown rice krispie treats[a]	Made with peanut butter from glass jars
**Beverages**	
Water	Provided stainless steel water bottle to each participant
Coffee	Instructed to use a French press or ceramic hand drip
Soda	Instructed to choose glass bottles or fountain drinks or to abstain from use
Milk	Advised to select milk in glass bottles

[a] Prepared by caterer from basic ingredients

Supplemental Material, Table 2. Urinary BPA and phthalate levels pre-intervention, during intervention and post-intervention
a) all subjects (n = 20)

		Creatinine (mg/dl)	BPA (ng/mL)	MEHP (ng/mL)	MEOHP (ng/mL)	MEHHP (ng/mL)	MEP (ng/mL)	MBUP (ng/mL)	MBZP (ng/mL)	MMEP (ng/mL)
Pre-intervention	Minimum	33	1	2.1	9.5	15	6.7	18	2.9	<MRL
	10th %ile	48	1.4	2.3	11	24	16	24	4	5.7
	25th %ile	60	1.9	3.4	13	28	25	30	4.6	6.8
	Median	100	3.4	4.5	17	42	34	39	8.3	13
	GM	94	3.7	7.1	27	57	41	43	12	12
	75th %ile	150	7.3	9.2	33	84	68	49	28	19
	90th %ile	190	11	41	88	200	110	80	34	27
	Maximum	220	16	190	630	1400	340	160	82	38
Intervention	Minimum	14	0.26	1.1	3.3	6.5	4.8	15	2.6	4.6
	10th %ile	38	0.47	1.3	4.8	11	12	15	4.1	6
	25th %ile	43	0.73	2.5	9.9	19	31	21	6.4	8.3
	Median	94	1.4	3.9	13	29	49	30	9.8	11
	GM	76	1.2	3.4	12	25	50	32	10	12
	75th %ile	130	2.1	5.5	17	37	110	44	20	16
	90th %ile	140	2.9	6.5	21	46	190	56	22	19
	Maximum	280	3.9	8.1	46	84	270	140	36	48
Post-intervention	Minimum	31	0.57	1.2	3.2	6.6	14	12	3.4	3
	10th %ile	35	1.1	1.4	6.3	15	15	24	5.9	5.9
	25th %ile	52	1.7	2.4	8.9	19	23	26	7.8	7.1
	Median	83	3.9	3.9	13	29	34	34	11	10
	GM	79	3.8	4.1	14	31	53	35	11	9.3
	75th %ile	130	8.4	6.7	25	53	150	47	18	13
	90th %ile	150	11	12	35	70	280	73	26	16
	Maximum	210	38	16	49	110	380	89	31	19

b) children (n = 10)

		Creatinine (mg/dl)	BPA (ng/mL)	MEHP (ng/mL)	MEOHP (ng/mL)	MEHHP (ng/mL)	MEP (ng/mL)	MBUP (ng/mL)	MBZP (ng/mL)	MMEP (ng/mL)
Pre-intervention	Minimum	33	1.2	2.1	11	15	6.7	34	3.9	5.7
	10th %ile	38	1.6	2.2	13	23	15	36	4.6	5.7
	25th %ile	51	1.8	2.3	13	25	16	42	6.1	7.9
	Median	68	2.6	3.9	16	35	25	46	12	15
	GM	70	3.3	4.1	21	42	21	53	14	13
	75th %ile	100	6	5.8	28	59	29	62	29	18
	90th %ile	120	12	8.5	60	120	32	81	57	21
	Maximum	160	16	15	66	150	39	160	82	38
Intervention	Minimum	14	0.26	1.1	3.3	6.5	4.8	15	2.6	4.6
	10th %ile	36	0.44	1.2	3.5	6.9	6.8	15	3.8	6.9
	25th %ile	40	0.52	1.4	6.3	14	16	18	4.5	7.6
	Median	45	1.2	2.6	13	25	33	29	11	14
	GM	52	1	2.4	12	23	27	29	9.3	13
	75th %ile	97	1.9	4.1	20	40	50	45	20	17
	90th %ile	120	2.8	4.5	26	49	70	56	22	30
	Maximum	130	2.9	5.4	46	84	110	58	22	48
Post-intervention	Minimum	31	0.57	1.2	3.2	6.6	14	18	4.2	3.7
	10th %ile	34	1	1.4	7	15	15	26	9.6	5.9
	25th %ile	36	1.3	2	9.1	20	16	28	11	7.7
	Median	56	3.3	2.9	19	34	26	38	19	11
	GM	59	3.1	3.4	15	30	29	38	16	10
	75th %ile	84	4.3	5.8	29	55	35	48	25	14
	90th %ile	110	12	9.1	34	60	58	73	26	16
	Maximum	140	38	11	37	70	220	74	31	19

c) adults (n = 10)

		Creatinine (mg/dl)	BPA (ng/mL)	MEHP (ng/mL)	MEOHP (ng/mL)	MEHHP (ng/mL)	MEP (ng/mL)	MBUP (ng/mL)	MBZP (ng/mL)	MMEP (ng/mL)
Pre-intervention	Minimum	58	1	3.3	9.5	22	32	18	2.9	<MRL
	10th %ile	61	1.4	3.4	11	28	34	19	3.9	5.6
	25th %ile	100	2.5	4	13	29	45	25	4.2	7.1
	Median	150	4.9	7.4	24	50	73	30	8	8.1
	GM	130	4.2	12	34	78	78	34	9.3	11
	75th %ile	180	8	28	36	89	97	36	21	19
	90th %ile	190	10	120	320	680	210	60	29	27
	Maximum	220	11	190	630	1400	340	160	32	34
Intervention	Minimum	36	0.54	2.5	7.5	16	30	19	4.5	6
	10th %ile	55	0.73	2.6	8.3	18	32	19	6.4	6
	25th %ile	88	1	3.2	11	23	43	22	6.8	8.8
	Median	130	1.6	5.2	13	30	110	33	8.5	11
	GM	110	1.5	4.7	13	29	92	35	11	10
	75th %ile	140	2.3	6.4	16	35	180	42	19	13
	90th %ile	170	3.2	7.2	17	40	240	64	24	16
	Maximum	280	3.9	8.1	20	53	270	140	36	18
Post-intervention	Minimum	45	1.1	1.3	6.1	14	23	12	3.4	3
	10th %ile	58	1.4	2.1	6.3	16	24	24	5.9	6.6
	25th %ile	80	2.3	3.4	8.7	18	43	26	6.5	7.3
	Median	130	5.9	4.4	9.9	28	110	31	8.1	8.9
	GM	110	4.6	4.9	13	32	98	32	8.3	8.7
	75th %ile	150	9	7.8	19	46	250	36	11	11
	90th %ile	160	11	16	36	76	290	63	14	14
	Maximum	210	14	16	49	110	380	89	17	17

BPA, bisphenol A; GM, geometric mean, MBuP, mono butyl phthalate (n-and iso); MBzP, mono benzyl phthalate; MEHP, mono-2-ethylhexyl phthalate; MEOHP, mono-(2-ethyl-5-oxohexyl) phthalate; MEHHP, mono-(2-ethyl-5-hydroxyhexyl) phthalate; MMeP, monomethyl phthalate; MRL, method reporting limit

Supplemental Material, Table 3. Self-reported dietary deviations during intervention

Family	Day of intervention	Reported deviation
1	1	Milk from #2 plastic container; one parent had coffee from machine
	2	None
	3	Bottle salad dressing
2	1	Dried noodles from foil pack; bread with butter and jelly from glass
	2	Crackers from foil-lined bag; eggs; ham from deli with edges touching packaging removed
	3	Ham from deli with edges touching packaging removed
3	1	None
	2	Peanut butter (glass jar) and jelly (glass jar) sandwiches
	3	Peanut butter (glass jar) and jelly (glass jar) sandwiches; milk from glass container; soy milk from tetra-pack container in coffee
4	1	None
	2	Bananas
	3	Chocolate milk
5	1	None
	2	None
	3	None

Supplemental Material, Table 4. Possible sources of BPA and phthalates before and after dietary intervention

		Average Consumption per Person per Day [a]			
Family	Time	Canned Foods	Restaurant Meals	Soda or Bottled Water [b]	Meals Microwaved in Plastic
1	pre	1	0.75	0.19	0
	post	0.25	1	0.5	0
2	pre	0.88	1.5	1	0
	post	0	0.5	1	0
3	pre	1.1	0.75	1	0
	post	0.33	1	1.7	0
4	pre	0.75	1	1.4	1
	post	1.7	0	1.7	0.67
5	pre	0.25	1.2	1.6	0.38
	post	0.17	0.58	0.33	0.83
Overall	pre	0.8	1.1	1	0.28
	post	0.48	0.62	1	0.3

[a] All values are rounded to 2 significant digits

[b] "bottled water" includes individual-serving plastic bottles, polycarbonate bottles, large polycarbonate coolers, and plastic filter pitchers

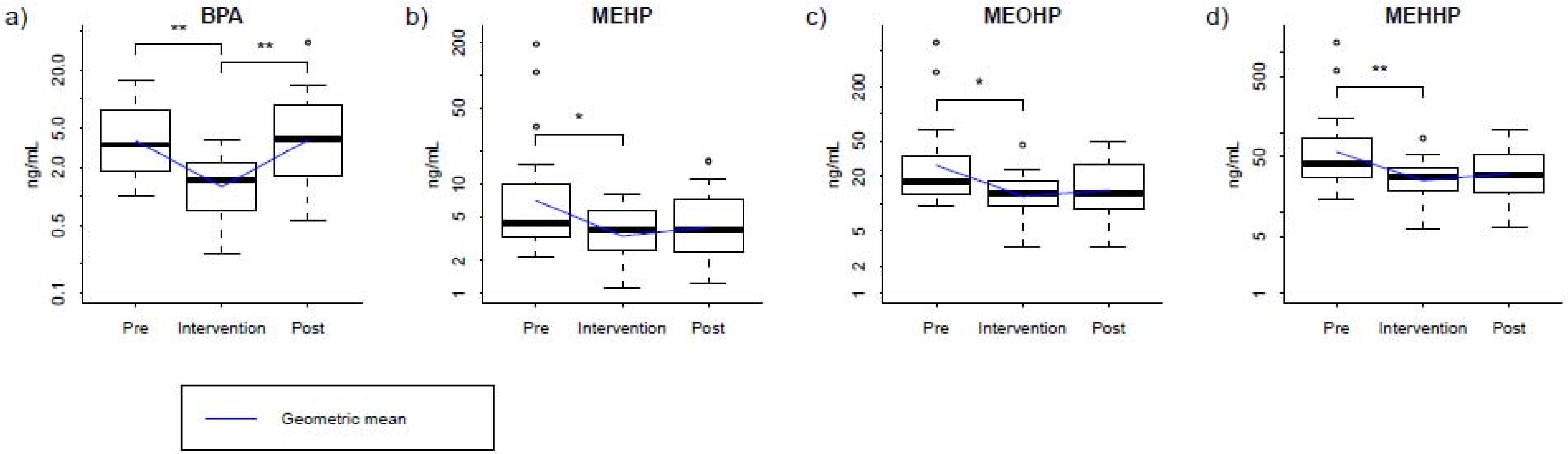

Supplemental Material, Figure 1. Boxplots of unadjusted urinary concentrations with Wilcoxon signed rank test results, Box and whiskers plots showing the distribution of unadjusted urinary levels of each analyte in the pre-intervention, intervention, and post-intervention samples. The fresh food intervention was associated with significant reductions in urinary excretion of bisphenol A (a) and metabolites of DEHP (b-d). Asterisks indicate significant differences between times as determined by Wilcoxon signed rank test: * indicates $p<0.05$; ** indicates $p<0.005$.

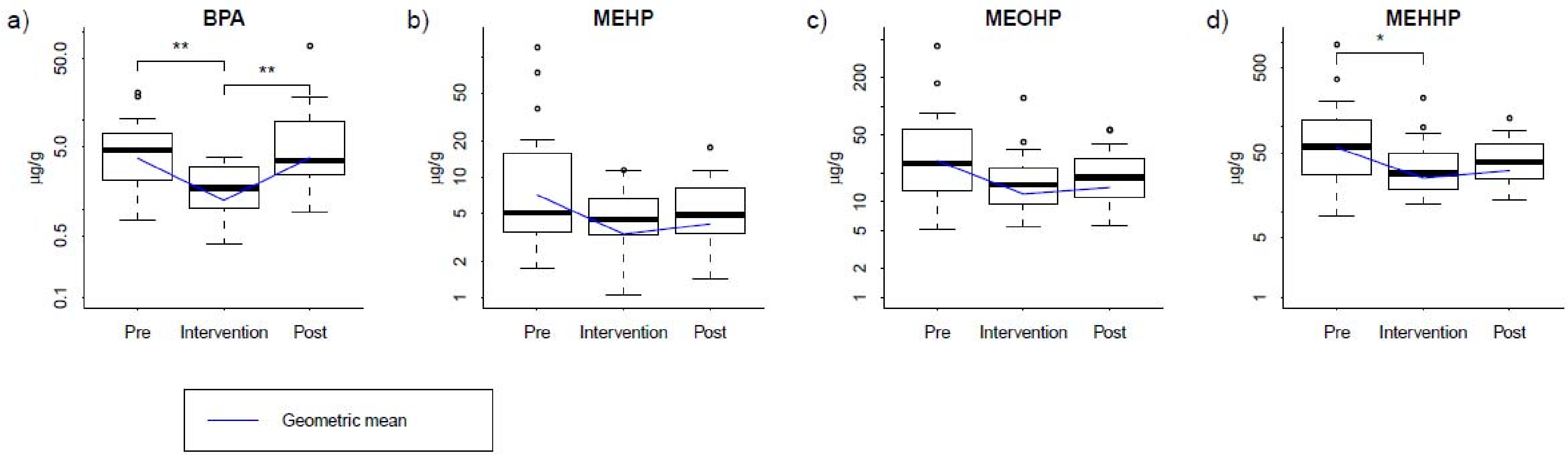

Supplemental Material Figure 2, Boxplots of creatinine-adjusted urinary concentrations with Wilcoxon signed rank test results. Box and whiskers plots showing the distribution of urinary levels, adjusted for creatinine, of each analyte in the pre-intervention, intervention, and post-intervention samples. The fresh food intervention was associated with reductions in urinary excretion of bisphenol A (a) and metabolites of DEHP (b-d), although the decrease was not statistically significant for creatinine-adjusted MEHP and MEOHP. Asterisks indicate significant differences between times as determined by Wilcoxon signed rank test: * indicates p<0.05; ** indicates p<0.005.

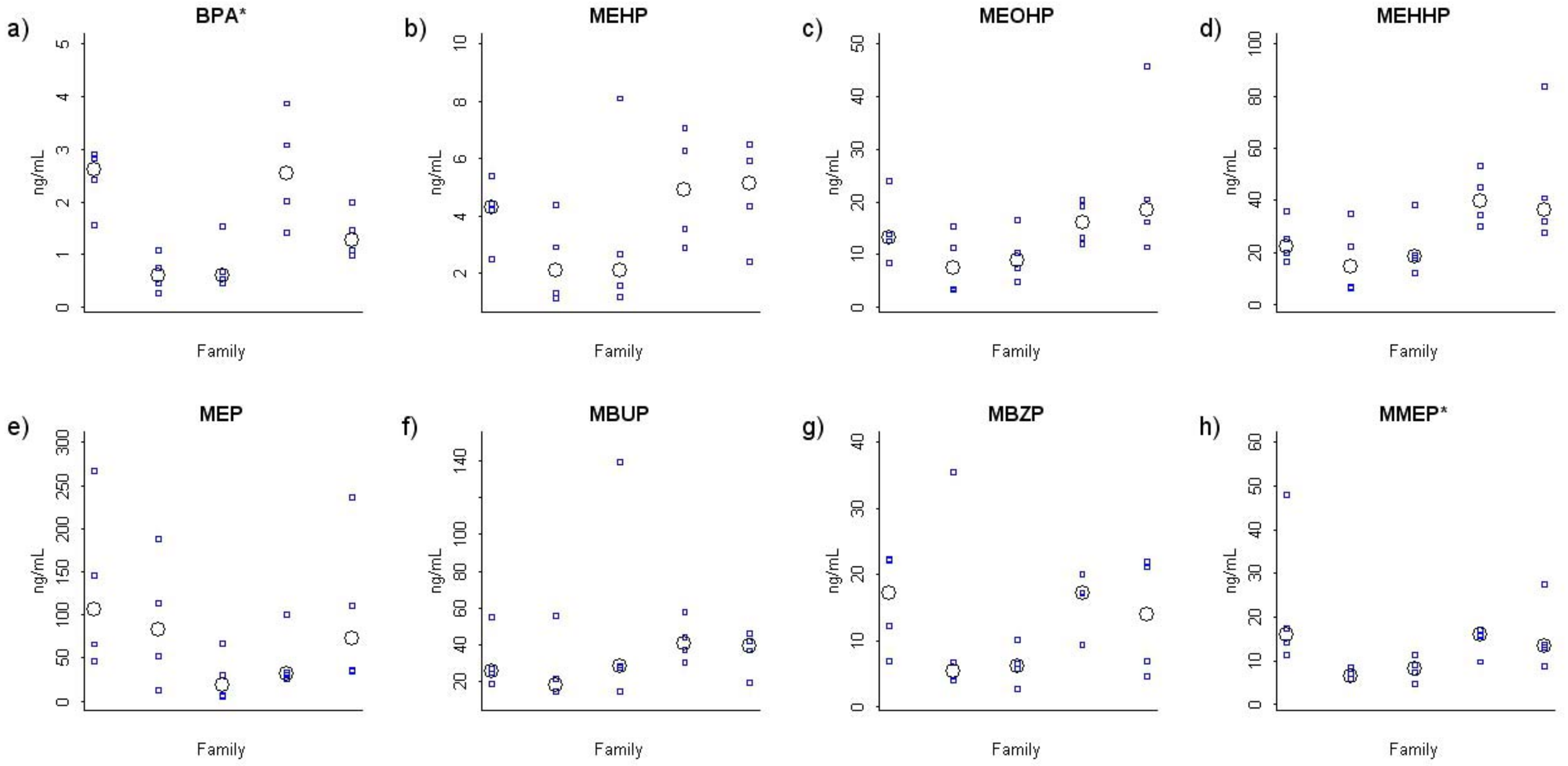

Supplemental Material Figure 3 Urinary levels of each analyte during dietary intervention, shown by family.
Strip plots showing the concentration of each analyte during the dietary intervention, grouped by family. Large circles indicate family medians. Asterisks next to compound name indicate significant variation between families, as determined by Kruskal–Wallis one-way analysis of variance by ranks: * indicates $p<0.05$.
